# Aurora B Tension Sensing Mechanisms in the Kinetochore Ensure Accurate Chromosome Segregation

**DOI:** 10.3390/ijms22168818

**Published:** 2021-08-16

**Authors:** Shelby L. McVey, Jenna K. Cosby, Natalie J. Nannas

**Affiliations:** Department of Biology, Hamilton College, 198 College Hill Road, Clinton, NY 13323, USA; smcvey@hamilton.edu (S.L.M.); jcosby@hamilton.edu (J.K.C.)

**Keywords:** chromosome segregation, Aurora B, spindle assembly checkpoint, kinetochore

## Abstract

The accurate segregation of chromosomes is essential for the survival of organisms and cells. Mistakes can lead to aneuploidy, tumorigenesis and congenital birth defects. The spindle assembly checkpoint ensures that chromosomes properly align on the spindle, with sister chromatids attached to microtubules from opposite poles. Here, we review how tension is used to identify and selectively destabilize incorrect attachments, and thus serves as a trigger of the spindle assembly checkpoint to ensure fidelity in chromosome segregation. Tension is generated on properly attached chromosomes as sister chromatids are pulled in opposing directions but resisted by centromeric cohesin. We discuss the role of the Aurora B kinase in tension-sensing and explore the current models for translating mechanical force into Aurora B-mediated biochemical signals that regulate correction of chromosome attachments to the spindle.

## 1. Introduction

**Figure 1:**
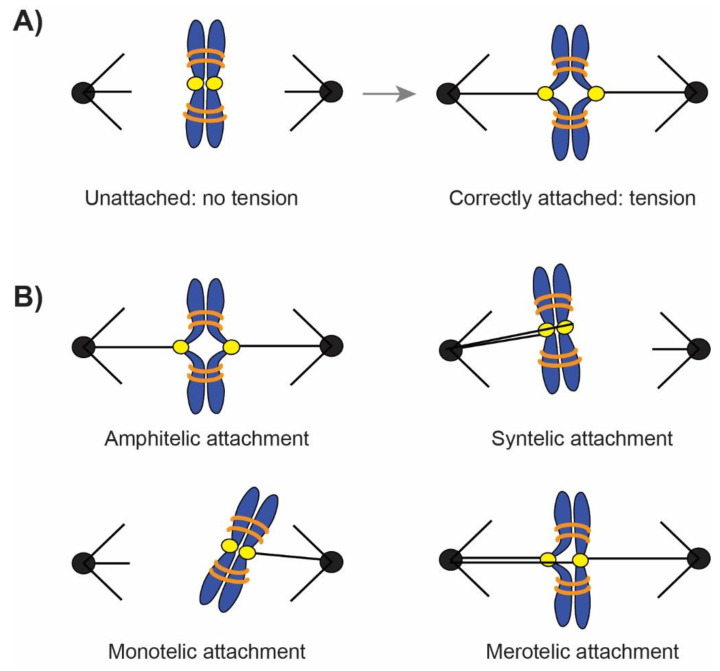
Attachment of chromosomes (blue) to the spindle (black) is facilitated by kinetochores (yellow). Sister chromatids are held together by cohesin (orange). (**A**) Unattached chromosomes experience no tension, but when they are correctly attached or bi-oriented, they experience tension force as the two chromatids are pulled apart by depolymerizing microtubules but resisted by cohesin. Both pericentric chromatin and kinetochores on bi-oriented chromosomes become visibly stretched under tension. (**B**) Correct attachments are also referred to as amphitelic attachments with kinetochores on sister chromatids attached to microtubules from opposite spindle poles. Incorrect attachments include: syntelic attachment where kinetochores on sister chromatids are attached to the same pole, monotelic attachment where only a single kinetochore is attached, and merotelic attachment where a kinetochore is attached to both poles. Incorrect attachments generate no tension (syntelic and monotelic) or improper tension (merotelic) on the chromosome.

Organisms package their genetic material into chromosomes; faithful segregation of these structures through cell division is critical as mistakes can lead to disease, infertility and congenital defects [1,2]. Eukaryotic chromosomes take on the characteristic X-shape after replication and chromatin condensation, consisting of two identical sister chromatids. Chromatids are separated in mitosis by the microtubule-based spindle apparatus. Attachment to the spindle occurs through the kinetochore, a large structure containing 80–100 proteins in sub complexes that are organized into inner and outer domains. The inner kinetochore assembles on centromeres through interactions with chromatin, and the outer kinetochore binds to spindle microtubules (for a review of kinetochore structure and function, see [3]). Depending on the species, approximately 1–30 microtubules attach per kinetochore. Budding yeast have the simplest architecture with a single kinetochore microtubule [4,5] while vertebrates average 15–20 microtubules/kinetochore [6]. To ensure dividing cells receive equal numbers of chromosomes, sister chromatids must bi-orient with their kinetochores attached to microtubules from opposite spindle poles (Figure 1A). Mistakes in attachment can occur: kinetochores can attach to microtubules from the same pole (syntelic attachment), a single kinetochore can attach to microtubules from both poles (merotelic attachment), or only one kinetochore can be attached (monotelic attachment) (Figure 1B). These errors must be corrected into the amphitelic configuration with all chromosomes achieving bi-orientation before anaphase. If not corrected, errors lead to chromosome segregation mistakes that result in aneuploidy. Meiotic aneuploidy is the most common cause of congenital birth defects and miscarriage [1] with approximately 10–30% of fertilized human eggs having an incorrect number of chromosomes [7]. Aneuploidy also remains the predominant cause of congenital birth defects [1]. Mitotic aneuploidy is linked to tumorigenesis [8], metastasis [9] and cancer cell motility [10]. The prognosis becomes more grim with increasing levels of aneuploidy as these cells are often less susceptible to chemotherapies [2].

The accuracy of chromosome segregation is protected by the spindle assembly checkpoint (SAC) which monitors kinetochore-microtubule attachments and activates in response to errors. The SAC delays anaphase until all chromosomes are bi-oriented on the spindle [11]. The checkpoint consists of many components originally identified in classical budding yeast genetic screens, including Mitotic Arrest Deficient (MAD) proteins [12], Budding Uninhibited by Benzimidazoles (BUB) proteins [13], the Mps1 kinase [14] and the cell cycle regulator Cdc20 [15,16]. Extensive work over the last decade has revealed that the checkpoint operates through a diffusible inhibitor initiating at kinetochores that blocks the Anaphase Promoting Complex (APC) [17,18]. Specifically, the main effector protein, Mad2, is recruited to kinetochores, where it catalyzes the formation of the Mitotic Checkpoint Complex that binds and sequesters Cdc20 from the APC. For a review of SAC components and function, see [19]. Delaying anaphase gives cells time to turn over incorrect attachments and generate correct attachments. The SAC is inactivated by correct attachments through a silencing network that displaces checkpoint proteins from the kinetochore [20,21]. Debate has surrounded the SAC trigger; some argue that SAC activation is caused by lack of tension on incorrect attachments and others argue activation is caused by unattached kinetochores [11]. The basis of this debate will be discussed below, but our review will focus on the role of tension in sensing and correcting improper attachments.

## 2. Tension Generation on Correct Attachments

Chromosomes that are correctly attached to the spindle experience mechanical pulling forces that generate tension across the sister chromatids (Figure 1A). Bi-oriented chromosomes are pulled in opposite directions during metaphase by depolymerizing microtubules, but separation is resisted by cohesin that holds the sister chromatids. Cohesin is a ring-shaped complex made up of Smc1, Smc3, Scc3 and Scc1 [22,23] with a diameter of approximately 30–35 nm that allows it to encircle the 10 nm packaged chromatin fibers of sister chromatids [24]. Enriched in centromeres and the surrounding 50 kb pericentric region [25], cohesin tethers replicated chromatids together until anaphase when the APC liberates the protease Separase that cleaves cohesin [26]. The force generated on chromosomes was first calculated by Bruce Nicklas in his pioneering work on grasshopper spermatocytes. Using force calibrated glass needles to impede chromosome movement; he estimated 700 pN of tension force is generated on each kinetochore [27]. Recent experiments have confirmed this estimate and extended it, finding that each kinetochore microtubule exerts approximately 15 pN of force [28]. Using FRET sensors, forces have been quantified to the molecular level with 0.45–2 pN of force experienced by single kinetochore protein molecules [29,30]. There are estimated differences across species, with *Drosophila* kinetochore microtubules exerting 12–62 pN of force each and thus their kinetochores experiencing 144–764 pN of tension [29]. Budding yeast kinetochores on the other hand experience 6pN of tension force [30].

The tension force generated on correctly attached sister chromatids stabilizes and increases kinetochore-microtubule attachments [31,32,33]. Nicklas demonstrated that artificially placed tension could stabilize incorrect kinetochore-microtubule attachments that would otherwise break down [27]. Increasing tension beyond physiological levels increases the number of stabilized kinetochore microtubules [31]. The ability to stabilize microtubule-kinetochore interactions is both direct (tension itself increases the association) and indirect (tension prevents destabilizing activity by other proteins). In vitro studies with reconstituted budding yeast kinetochores demonstrated that tension directly increases the lifetime of kinetochore-microtubule attachments [32]. The association time matches in vivo imaging of single microtubules interacting with kinetochores under tension [34]. The relationship between kinetochores and microtubules thus demonstrates a ‘catch bond’ interaction seen with other biological molecules where tension force decreases disassociation rates [32,35,36]. Tension also indirectly promotes microtubule-kinetochore interaction by preventing the Aurora B kinase from phosphorylating kinetochore proteins and causing a release of the attachment. Aurora B regulates many aspects of mitosis [37], but it plays a critical role in sensing the tension-state of chromosome attachments by recognizing and selectively destabilizing incorrect attachments that do not generate tension [38].

## 3. Tension vs. Attachment: Activation of the Spindle Assembly Checkpoint

Historically, mechanistic explanations of the SAC activation by mis-attached chromosomes have fallen into two competing camps: one stating that unattached kinetochores trigger the SAC, and the other pointing to lack of tension on kinetochores. The tension model was originally based on Nicklas’ manipulation of force on chromosomes. In addition to demonstrating that tension stabilized attachments, Nicklas also showed that exerting tension on incorrectly attached chromosomes induced metaphase-arrested cells to enter anaphase [39]. In contrast, Conly Rieder and colleagues concluded that kinetochore attachment status is the checkpoint trigger. Conley showed that laser-ablation of the last unattached kinetochore stimulated cells to enter anaphase [40]. Ablation would not have created tension but rather eliminated the presence of an unattached kinetochore, arguing for an attachment status trigger. These opposing studies established the two models of SAC activation, and recent work has continued the debate with supporting evidence for both models. Distinguishing between the models is complicated by Aurora B, as this kinase displays activities important for both models. It generates unattached kinetochores by turning over incorrect attachments [41,42] but it is responsive to the tension state on chromosomes [43,44,45].

Studies in budding yeast have provided a simplified approach to tension vs attachment. With a single microtubule attaching per kinetochore, budding yeast have binary attachment status that eliminates the complication of multiple microtubules attachments per kinetochore [46]. Tensionless attachments can be generated in yeast by inhibiting replication or cohesin [47]; these attachments stimulate the SAC to delay anaphase, and this arrest is dependent on the yeast Aurora B homolog [43]. Recent studies also support the role of tension in SAC activation and error correction; Mukerjee et al. demonstrated that cells are sensitive to the magnitude of tension. By creating a gradient of tension, they observed a similarly graded response in phosphorylation levels, unattached kinetochores, segregation errors, and metaphase delay time [45]. Similarly, Chen et al. found that the outcomes of Aurora B phosphorylation is tension-dependent, with different biochemical responses to high and low tension [35]. The SAC is also activated in response to manipulation of tension that leaves attachments intact but reduces tension such as taxol treatment [48] and manipulating motor proteins [49,50]. Tension has been similarly demonstrated to be critical in meiosis, where SAC activation is responsive to lack of tension on bivalent chromosomes in meiosis I but not responsive to attachment status [50].

Recent studies have also supported tension-independent pathways regulating SAC activation. Borealin, a member of the Chromosome Passenger Complex involved in Aurora B localization, was shown to regulate SAC activity independent of tension. Disruption of a microtubule-binding domain in Borealin resulted in increased attachment errors, disrupted SAC signaling, and decreased phosphorylation of incorrect attachments regardless of tension state [51]. In other studies, monopolar spindles incapable of creating tension-generating attachments were able to satisfy and silence the SAC, showing that cells may verify incorrect attachments without tension signals [52,53]. Additionally, studies showed that only a minimal number of attachments were sufficient to satisfy the SAC. In mammalian cells where ~20 microtubules attach per kinetochore, only four microtubule attachments were necessary to silence SAC activity on the kinetochore despite the reduced tension generated [54].

**Figure 2:**
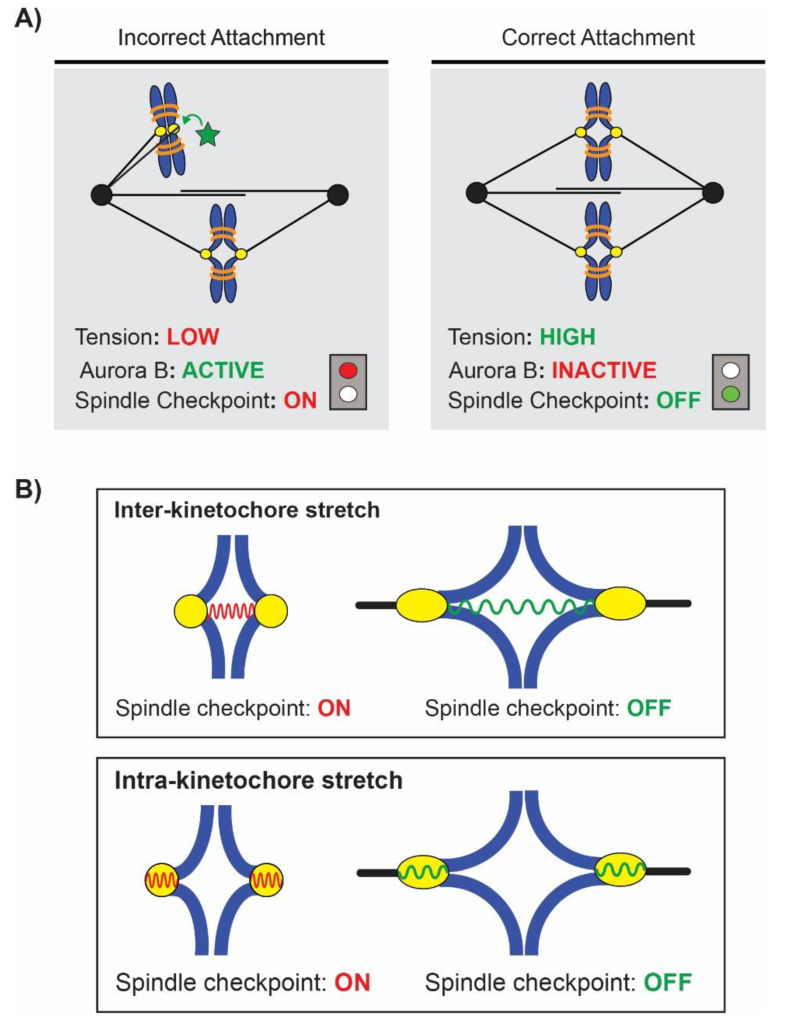
The spindle assembly checkpoint is activated by lack of or low tension on chromosomes. (**A**) Incorrect attachments have no or low tension, Aurora B kinase is active (green star) and the checkpoint is activated, halting cell cycle progression in metaphase until the error can be corrected. Correct attachment generates high tension, Aurora B kinase is inactive, and satisfies the checkpoint, silencing it and allowing the cell to proceed to anaphase. (**B**) Tension has been proposed to be measured either in the inter-kinetochore or intra-kinetochore region. Inter-kinetochore stretch is the distance between sister kinetochores and is facilitated via the stretching of spring-like elastic pericentric chromatin that inactivates the checkpoint. Intra-kinetochore stretch is the deformational distance produced within a kinetochore under tension and is facilitated via stretching and conformational change of kinetochore proteins.

Distinguishing between tension and attachment in SAC activation and error correction is complicated by their inter-relationship. As described above, the two have a positive feedback relationship wherein tension stabilizes and promotes attachment, leading to increased attachments [27,31,32]. Aurora B activity unites these two models as it responds to lack of tension by both creating unattached kinetochores that trigger the checkpoint and by directly activating the checkpoint [55,56]. Regardless of the SAC trigger debate, tension has been definitively shown to serve as a signal of correct kinetochore-microtubule attachment [45,47] and stimulate Aurora B activity in its absence [44,57]. Chromosomes that are correctly attached experience high levels of tension that satisfies and silences the SAC; chromosomes that are incorrectly attached experience low tension, which is detected by Aurora B and leads to activation of the SAC and arrest in metaphase (Figure 2A).

## 4. Where Is Tension Sensed? Inter-Kinetochore vs. Intra-Kinetochore Stretch

Tension regulates the SAC and the correction of erroneous attachments, but the location of tension-sensing is still unclear. When sister chromatids are correctly attached to opposite poles, both kinetochores [58,59,60] and the pericentric chromatin [34,61,62] undergo conformational deformation under tension (Figure 1A). These two locations have each been proposed as the site monitored by Aurora B for tension status, either the stretch on chromatin and thus the separation of sister kinetochores (inter-kinetochore stretch) or stretch within an individual kinetochore (intra-kinetochore stretch) (Figure 2B). Support for inter-kinetochore tension-sensing is based on the large degree of chromatin stretch and separation of sister kinetochores visualized on chromosomes under tension. In budding yeast, GFP-labelled pericentric chromatin stretches up to 1µm during metaphase, approximately half of its 2 µm spindle [63]. Chromatin stretch is dynamic, and has been visualized undergoing cycles of stretching and retracting [61,62,64] matching the dynamic instability of microtubules. Measurements of pericentric chromatin dynamics has shown that this region behaves with elastic, spring-like properties [65,66,67,68]. These spring properties are established through a series of intramolecular loops in the pericentric chromatin [69]. Inhibiting microtubule dynamics with BAL27862, a novel microtubule-destabilizing drug that disrupts kinetochore attachments and microtubule organization [70], causes a reduction in inter-kinetochore stretch distance and generates a slight delay in anaphase in human RPE1 cells [71]. However, the study concluded that inter-kinetochore stretch was not critical as chromosomes with no kinetochore separation still satisfied the SAC and entered anaphase [71]. Similarly, when inter-kinetochore stretch is eliminated by tethering pericentric chromatin together, budding yeast cells do not activate the SAC and enter anaphase with wildtype timing [72].

Kinetochores have been shown to elongate under tension and this intra-kinetochore stretch is the other proposed site of tension-sensing [58,59,73,74]. By fluorescently tagging various kinetochore proteins, several studies have demonstrated deformation and structural rearrangement within the kinetochore under tension. The distance between inner kinetochore protein CENP-C and outer components Ndc80 and Cdc20 changed by up to 15% or 20 nm in Ptk2 cells [60]. In human RPE1 cells, stretching between inner (CENP-A) and outer (Ndc80) components increases throughout prometaphase with 40% of kinetochores visibly stretched in metaphase [74]. The outer kinetochore experiences reorganization under tension via a jackknifing action of the Ndc80 protein, unraveling of the Knl1 protein [75], and extension of the unstructured domain of CENP-T [74]. Recent microscopy and modeling of CenpA-Ndc80 distances in different species suggests that at least 50% of intra-kinetochore distance changes is caused by malleable reorganization of proteins as well as elastic stretching of proteins [76]. Some of the strongest evidence for measurement of intra-kinetochore distance comes from studies that uncouple the two types of stretch. When treated with taxol, *Drosophila S2* cells display reduced inter-kinetochore stretch but not intra-kinetochore stretch; the cells did not activate the checkpoint [59]. Similarly, the checkpoint did activate in response to reduced intra-kinetochore stretch in HeLa cells treated with low doses of nocodazole; inter-kinetochores stretch was not impacted by the nocodazole treatment. Inter-kinetochore stretch has recently been shown to facilitate silencing of the checkpoint by promoting the recruitment and elimination of different signaling proteins at the kinetochore [74].

## 5. Responding to Tension: Aurora B Kinase

The Aurora B kinase is the critical regulator of tension-based response to chromosome attachments. Originally identified in a budding yeast screen to identify genes involved in the regulation of chromosome segregation, Aurora B (Ipl1 in yeast) was responsible for changes in ploidy [77]. Without Aurora B, incorrect attachments persist [43,78,79], and result in chromosome segregation errors [80]. As a serine/threonine kinase with a [RK]x[TS][ILV] consensus sequence [81,82], Aurora B phosphorylates components of the outer kinetochore in response to lack of tension. Phosphorylation of these targets destabilizes incorrect attachments and promotes the establishment of correct attachments [32,41,44,81]. The kinase’s localization, activation, and pattern of phosphorylation are all critical to its ability to regulate chromosome attachment.

### 5.1. Aurora B and the Chromosome Passenger Complex

Aurora B is regulated and localized on chromosomes via the chromosome passenger complex (CPC) consisting of three non-enzymatic subunits: Borealin, Survivin and INCENP [83]. Borealin is critical for CPC localization; when Borealin-nucleosome interactions are hindered, the CPC is not recruited to chromosomes and results in misalignment [84,85]. Survivin is essential for the recruitment of Aurora B to the CPC, and may assist SAC silencing. In cells lacking functional Survivin, the time from chromosomes alignment to anaphase onset was significantly longer [86]. Survivin is recruited to centromeric chromatin via Haspin-mediated phosphorylation of histone H3 [87,88]. INCENP is the main scaffold protein stabilizing the CPC complex [89] that is also localized via H3 phosphorylation [87]. Its N-terminus forms a three helix bundle with Survivin and Borealin [90], while its C-terminus contains an IN-Box domain critical for binding and activating Aurora B [91,92]. The internal domain of INCENP forms a single alpha helix that binds microtubules [93,94] and forms a bridge to centromeric chromatin [95]. Without functional INCENP, proper CPC localization, outer kinetochore assembly, and activation of Aurora B are all inhibited [96]. In *Drosophila* meiosis, point mutations in the IN-Box cause increased segregation errors, including early separation of sister chromatids in meiosis I [97].

Localization of Aurora B and the CPC is critical for proper chromosome segregation. During prometaphase and metaphase, the CPC localizes at the inner centromere, then relocates to the spindle midzone in anaphase [98] where it plays a role in mitotic exit [99]. Studies in HeLa demonstrated that precise inner centromere localization of the CPC is critical for SAC silencing and preventing premature separation of sister chromatids [100]. However, precise CPC localization was not required for Aurora B correction of erroneous attachments [100]. This separation of function based on localization is supported by recent studies that identified multiple pools of Aurora B. Distinct populations were found localized to the inner centromere, outer centromere proximal to kinetochores, and the outer kinetochore [86,101,102]. Each population was uniquely recruited (see “Kinetochore localization model” section for details) and demonstrates separation functions. The two chromatin-based pools appear to regulate SAC silencing in response to tension, but do not contribute to phosphorylation of erroneous attachments [86,101,102]. These studies suggest that the kinetochore-based population of Aurora B/CPC may be the pool critical for destabilizing incorrect attachments.

### 5.2. Targets and Consequences of Aurora B Phosphorylation

Aurora B targets multiple proteins for phosphorylation in response to erroneous attachments including kinetochore proteins, CPC members, and spindle checkpoint components. Phosphorylation of outer kinetochore proteins in the KMN network (Knl1, Mis12 and Ndc80) triggers a release of microtubule attachment [41,44]. The unstructured N-terminal tail of Ndc80 is positively charged [103] and directly interacts with negatively charged microtubules, stabilizing their polymerizing tips [33]. Deletion of this N-terminal tail results in 100× reduced affinity for microtubules in humans [103] and 10× reduction in yeast [104], but does not impact affinity in *C. elegans* [105]. Phosphorylation reduces the tail’s positive charge, decreasing the affinity for microtubules and causing a release of the attachment [41,103,106,107] as well as stimulating microtubule depolymerization [33]. Aurora B phosphorylates other members of the KMN network, targeting the N-terminus of Knl1 and the Mis12 complex subunit Dsn1 [44,108]. Phosphorylation of these proteins sensitizes activity of the KMN network, producing a graded response that allows for fine-tuned regulation of microtubule interaction. Aurora B also phosphorylates kinetochore protein CENP-C, which assists error correction by disrupting Mis12 location and interactions between the inner and outer kinetochore [109]. While not a kinetochore protein, Aurora B-phosphorylation of the centromere-associated, microtubule-depolymerizing kinesin MCAK impacts kinetochore attachments as well [110,111]. Phosphorylation changes the kinesin’s conformation, reducing its microtubule affinity and depolymerization activity [112].

Aurora B has also been shown to phosphorylate CPC members: INCENP and itself. Phosphorylation of INCENP by Aurora B affects its ability to bind microtubules, which impairs its migration to the spindle midzone in anaphase [94,113]. Studies in *C.elegans* and HeLa cells have also shown that Aurora B phosphorylation of the C-terminus of INCENP stimulates greater kinase activity, resulting in a positive feedback loop that reinforces kinase activity at kinetochores [114,115]. Aurora B also auto-phosphorylates, and this activity aids its localization [116], its mitotic exit function [117] and accurate chromosome segregation [118].

Aurora B promotes the activation and maintenance of the SAC signaling. Inhibition of Aurora B through ZM447439 drug treatment prevented the kinetochore localization of SAC proteins BubR1, Mad2, and CENP-E [119]. Additionally, Aurora B inhibition prevented BubR1 phosphorylation, necessary for SAC function and chromosome alignment [119]. *Xenopus* immunodepletion assays showed that Aurora B also controls the localization of checkpoint proteins Mps1, Bub1 and Bub3, which inhibits downstream SAC signaling and complex formation [120]. More recent studies have shown that Aurora B enhances the localization of Mps1 and BubR1 to maintain checkpoint signaling [121] as well as directly activating the SAC through Mps1 [55].

## 6. Aurora B Tension-Sensing Models

Many models have been proposed to explain the Aurora B-based translation of mechanical tension into biochemical signaling, and they generally group into three categories: (1) spatial separation models, where Aurora B is physically separated from its targets by tension, (2) tension-sensitive activation models, where the activity of Aurora B is modulated by another protein in response to tension, and (3) kinetochore localization models, where a subpopulation of Aurora B localizes on kinetochores in early mitosis when tension is low, and (4) a new model in which the downstream effects of Aurora B phosphorylation are differentiated by tension rather initial sensing by the kinase.

### 6.1. Spatial Separation Models

Spatial separation models are based on the localization of Aurora B within the inner centromere where it is proposed that the kinase remains constitutively active after initial activation [122]. Under low tension and thus little inter- or intra-kinetochore stretch, Aurora B can reach its targets in the outer kinetochore, phosphorylating and turning over incorrect attachments. However, when correct attachments are generated, the outer kinetochore is stretched away from Aurora B, physically separating the kinase from its targets [57]. This model was first established in a study by Liu and colleagues using FRET-based assays to show that the kinase can phosphorylate targets in the centromere but not the outer kinetochore under tension. Eliminating tension allows the kinase to reach targets in the outer kinetochore. Additionally, artificial localization of the kinase to the outer kinetochore caused destabilization of all attachments [57]. In support of this model, it was shown that the position of proteins within the kinetochore impacts the timing of their phosphorylation [44,123]. When correct attachments are generated, phosphorylation is first reduced on the most outer kinetochore proteins and followed by progressively internal proteins [44]. The spatial separation model has been challenged by experiments that show inner centromere localization is not critical. In a budding yeast study, truncations of INCENP displaced Aurora B from the inner centromere but the kinase was still able to support proper tension-sensing and chromosome segregation [124].

#### 6.1.1. Spatial Separation: Dog Leash Model

**Figure 3:**
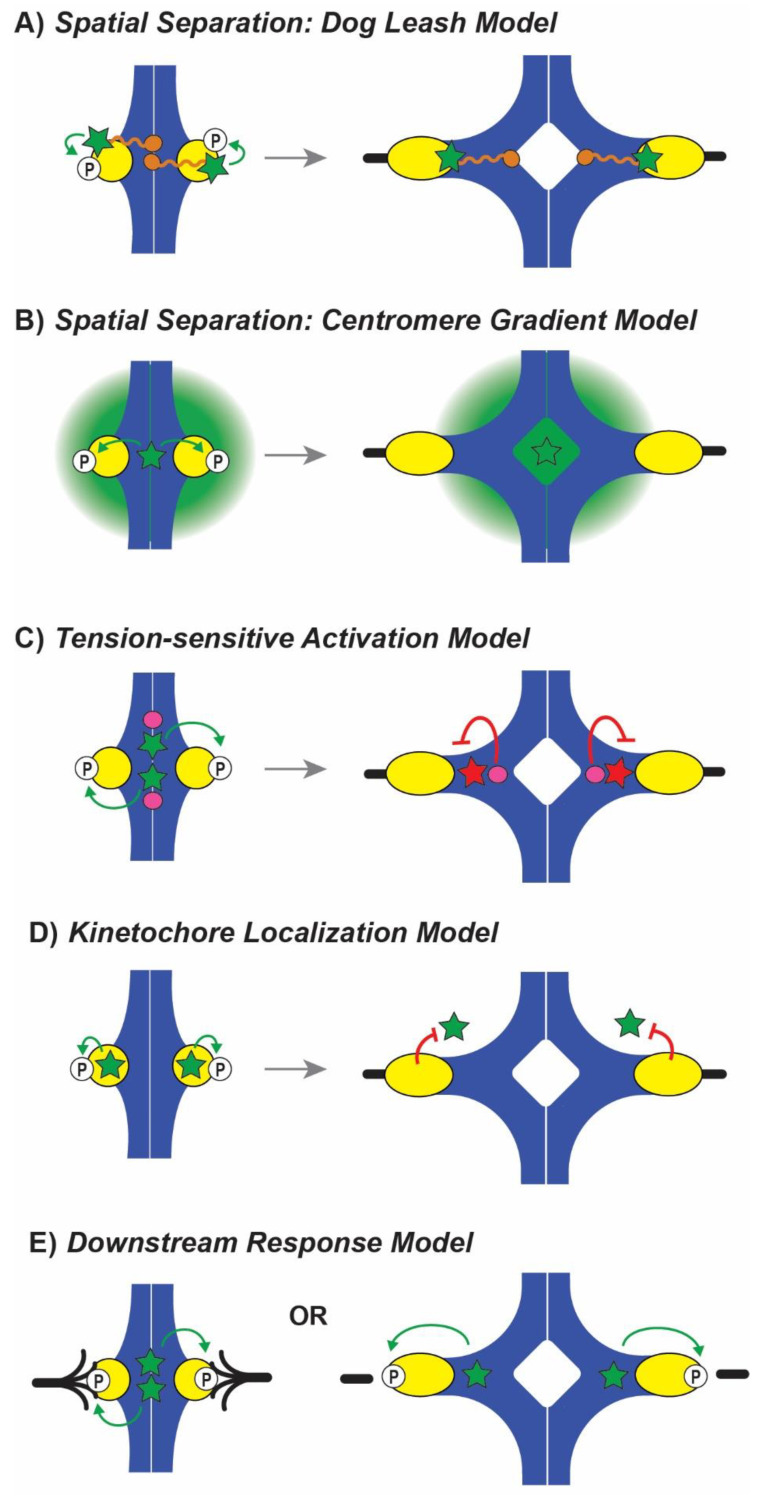
Current models of Aurora-B tension-sensing mechanisms. Chromatids (blue) and bound by kinetochores (yellow) are regulated by Aurora B (star) based on tension. Attachments to microtubules (black line) are disrupted if attachments lack tension, but maintained if they generate tension. (**A**) In the dog leash spatial separation model, Aurora B is tethered by leash protein INCENP (orange) that allows the kinase to reach and phosphorylate targets in the outer kinetochore when tension is lacking but not when tension is high. The kinase remains active (green) in both tension and tension-less states, but cannot reach the outer kinetochore due to stretch distances. (**B**) Similar to the dog leash model, the centromere gradient model proposes a constitutively active Aurora B kinase (green star) that is able to phosphorylate targets when tension is low, but targets are stretched beyond the gradient of Aurora B activity under tension. (**C**) Tension-sensitive models posit that Aurora B activity or action is differentially regulated by another protein (pink) in response to tension. When tension is low, the regulating protein activates (or does not suppress) Aurora B, and when tension is high, kinase activity is suppressed (red star). (**D**) Kinetochore localization models propose that the pool of Aurora B responsible for error correction is located within the kinetochore, phosphorylating targets under low tension, but the kinase is displaced from the kinetochore under high tension. (**E**) Downstream response model differs from other models because it focuses on the consequence of Aurora B phosphorylation rather than the tension-based regulation of kinase activity. If an attachment lacks tension, phosphorylation will stimulate microtubule depolymerization while still maintaining attachment to the kinetochore. However, if the attachment is under tension, phosphorylation will result in a release of the microtubule attachment.

Recent studies have yielded two variations of the spatial separation model: the dog leash model and the centromere gradient model [122]. The dog leash model (Figure 3A) proposes that spatial separation of Aurora B is facilitated by INCENP which acts as a leash that tethers the kinase to a restricted region [94]. In DT40 cells, the INCENP “leash” is estimated to be 80 nm long, and when truncated, phosphorylation of Dsn1 decreases while inner centromere phosphorylation (H3Ser28) remains constant. Truncations of INCENP in human cells also reduces the phosphorylation of outer kinetochore proteins Dsn1, KNL1 and Hec1 but not inner centromere protein CENP-A or chromatin [125]. Together, these studies suggest that INCENP tethers Aurora B to a region specified by its length, and under tension, the INCENP leash prevents the kinase from reaching the outer kinetochore.

#### 6.1.2. Spatial Separation: Centromere Gradient Model

An alternative spatial separation model is based on a gradient of Aurora B where kinase activity is high near the inner centromere (Figure 3B). When tension is high, the outer kinetochore stretches away from the zone of active Aurora B [122]. It has been proposed that Aurora B is activated by centromere-localized CPC members then released as a diffusible unit that quickly loses activity [126]. Supporting this model is the high turnover of CPC members at the inner centromere with half-lives of less than 1 min [127,128]. The dynamics of Aurora B flux at the centromere can be manipulated, resulting in altered phosphorylation patterns [129]. Additionally, recent studies have found dose-dependent responses to lack of tension rather than a binary response that might be predicted by a leash model. Mukherjee et al. found that a graded reduction of tension through kinesin-5 manipulation resulted in a similarly scaled response in kinetochore detachment and chromosome mis-segregation explained by Aurora B phosphorylation of outer kinetochore protein Dam1 [45]. One argument against this model is the steep gradient required. Previously measured Aurora B gradients that organize and position spindles in anaphase operate over micrometer lengths [98,130] but this model requires an activity boundary within 100 nm to prevent outer kinetochore phosphorylation [38,122].

### 6.2. Tension-Sensitive Activation Models

In contrast to spatial separation models where an active Aurora B kinase cannot reach its targets, tension-sensitive models posit that the activity of Aurora B is regulated by tension. This regulation can occur via a “tensiometer”, a spring-like protein that alters Aurora B activity, or via inhibitory proteins that counteract Aurora B. Several proteins have been proposed to act as a spring regulator including INCENP [95] due to its required presence for Aurora B activity [91,92], the conformational change of its internal SAH domain under tension [94], and the importance of this domain for mitotic progression [125]. PICH, a DNA translocase, has been proposed to measure inter-kinetochore stretch to regulate Aurora B due to its appearance as long threads connecting sister kinetochores [131,132]. Deletion of PICH in avian cells causes chromosome mis-segregation and chromatid non-disjunction [133]. Another potential regulator is CENP-E, a kinesin-7 motor protein that localizes on kinetochores and assists with chromosome alignment [134]. Using Single-Molecule High-Resolution Co-localization microscopy, Taveras et al. showed that CENP-E undergoes structural rearrangement under tension, specifically in its flexible coiled-coil domain [49]. Truncating this domain disrupts the conformational stretch and results in increased Aurora B phosphorylation of Ndc80 and chromosome misalignment [49].

Aurora B regulation in response to tension has also been proposed through balance of inhibitory activities. Protein phosphatases PP1 and PP2A localize to the outer kinetochore [135], with PP2A specifically enriched on unattached kinetochore [136]. PP2A interacts with kinetochore proteins specifically through a conserved docking motif LxxIxE identified on many proteins including BubR1 [137]. These serine/threonine phosphatases counteract Aurora B phosphorylation to stabilize attachments, and promote bi-orientation [136,138,139]. Their activity is tied to declining cyclin B kinase levels in late mitosis [140,141] that leads to physical interaction and reciprocal activation of the two phosphatase [142]. PP2A activity is opposed by the phosphatase inhibitor SET/TAF1 that localizes at the inner centromere [143] and decreases in abundance away from the centromere [144]. Overexpression [143] and ectopic kinetochore localization [144] of SET/TAF1 results in expanded zones of Aurora B activity. The activity and localization of PP2A and SET may be tied to mitotic stage, changing from early prometaphase when tension is low to late metaphase when tension is high [139,143,144]. The timing of phosphatase activity at the kinetochore is critical for the proper mitotic progression. PP1 binds a basic patch in the N-terminus of kinetochore protein Spc105/Knl1, and this interaction is critical for silencing the spindle checkpoint, however pre-mature interaction of PP1 with the kinetochore protein before bi-orientation interferes with error correction activities [145].

### 6.3. Kinetochore Localization Model

Recent studies on Aurora B localization has revealed three distinct pools of the kinase [86,101,102]. Haspin phosphorylation of histone 3-Thr3 recruits Aurora B and CPC members to the previously identified inner centromere location, while Bub1 phosphorylation of histone 2A-Thr120 recruits Aurora B to a distinct chromatin location in the outer centromere, proximal to kinetochores [86,101,102]. In addition to these chromatin locations, Broad et al. identified a third pool of Aurora B located within the kinetochore, between CENP-C and the N-terminus of Ndc80 that is not dependent on chromatin phosphorylation [102]. Earlier studies had identified a kinetochore-based pool of Aurora B as well, specifically a pool localized near CENP-A [146]. This was pool was distinguished by its phosphorylation state; kinetochore-localized Aurora B is phosphorylated at serine 331 via the Chk1 kinase whereas staining for the total Aurora B population showed localization extended along the centromere [146]. The kinetochore- and chromatin-based Aurora B populations appear to play distinct roles: inhibition of Haspin and Bub1 does not disrupt outer kinetochore phosphorylation activity [101,102] or fidelity of chromosome segregation but does impact silencing of the checkpoint [86]. The uniquely phosphorylated kinetochore-based pool is critical for accurate segregation and mitotic exit. Overexpression of an Aurora B S331A mutant causes spontaneous mis-segregation and impaired checkpoint function when challenged with taxol [146] whereas the phosphomimetic S331E mutation rescues chromosome alignment and segregation [147].These results suggest that the kinetochore-based Aurora B population is responsible for responding to incorrect attachments, calls into question spatial separation models as this population may not be separated from targets under tension [38,101,102].

A new model has been proposed based on kinetochore-localized Aurora B in which the kinase is directly recruited to kinetochores where it phosphorylates targets until it is displaced by tension, either through conformational change of binding sites or by active eviction [38]. Remaining questions for this model include identifying Aurora B-kinetochore binding sites and responsible recruiting proteins, as well as outlining the mechanism for tension-based displacement. There is support for Aurora B recruitment by kinetochore proteins; in yeast, the COMA kinetochore sub-complex has been shown to physically interact with INCENP and recruit Aurora B to inner kinetochores independently of Survivin and other CPC members on chromatin [123]. Additionally, the kinetochore protein Knl1 is required for Aurora B activity [148] and Knl1 has been shown recently to change structural conformation under tension [75], acting perhaps as a binding regulator of Aurora B [38,102].

### 6.4. Downstream Response Model

The models outlined above are based on the premise that Aurora B is responsive to tension, either through changing localization (spatial separation and kinetochore localization models) or altering kinase activity (tension-sensitive models). A recent study by Chen et al. proposes an entirely different mechanism; rather than differential Aurora B action based on tension, the authors propose a model in which the downstream effect of Aurora B phosphorylation is different depending on the tension-state of the attachment [35]. Aurora B phosphorylation had been shown to cause both release of microtubules due to reduced binding affinity [149,150] or depolymerization of microtubules without kinetochore release [33,151]. Chen et al. demonstrated that the tension-state of the attachment determines which of these two responses occurs in vivo.

Active Aurora B was transiently targeted to specific kinetochores using an optogenetics approach. The outer kinetochore protein Spc25 and the Aurora B- activating domain of INCENP were tagged with light-sensitive dimerizing domains; when activated, a photo-caged ligand was released and the two domains transiently heterodimerized [35]. This approach ectopically localized active Aurora B on individual kinetochores, both those with amphitelic attachments under tension and those with syntelic attachments lacking tension. By imaging live cellular responses, Chen et al. showed that phosphorylation of correct attachments caused a release of microtubules, while phosphorylation of syntelic attachments caused depolymerization of microtubules while maintaining the attachment [35]. Release could also be induced on syntelic attachments, but it required doubling the amount of tethered active Aurora B.

This study demonstrates that the biochemical response to phosphorylation depends on the mechanical tension-state of the attachment. Kinetochores typically exhibit a catch-bond interaction with microtubules, where tension stabilizes the interaction and increases the disassociation time [32,36]. Chen et al. showed that the catch-bond interaction is converted to a slip-bond by phosphorylation, where tension destabilizes the interaction, decreases the disassociation time, and causes a release event [35]. This model provides a mechanistic explanation of how cells can provide differential responses to syntelic and merotelic attachments. Merotelic attachments, while incorrect and potentially damaging to a cell, still generate tension and must be corrected. In this downstream response model, Aurora B would phosphorylate these attachments and release would occur due to the high tension. Detachment would leave the chromosome in the spindle midzone to become correctly attached. Syntelic attachments however are connected to the same pole and are often caused by the location or orientation of the chromosome. Phosphorylation-induced depolymerization would cause poleward retraction of the chromosome where polar ejection forces could then push it back towards the midzone for correct attachment. The major outstanding question for this model is how Aurora B is silenced on correct attachments.

## 7. Conclusions and Future Directions

Decades of research has yielded great advancements in the understanding of chromosome segregation. The proper attachment of chromosomes to the mitotic spindle and the ability to correct errors is critical for an organism’s viability. The role of tension has been firmly established as a mechanism to distinguish between correct and incorrect attachments. The Aurora B kinase is the main effector of tension-based corrections, and several models exist to explain how it senses tension. Spatial separation of the kinase from its targets or tension-based regulation of kinase activity are two firmly established models, but recent studies have yielded two new models. A kinetochore localization model posits that a subpopulation of Aurora B located on kinetochores is responsible for tension-based error correction [102], and a model based on downstream effects of phosphorylation argues that tension itself determines the mechanism of correction [35].

A critical consideration for all of these models is that they need not be mutually exclusive, and in fact, a combination of models could address many outstanding questions. While Chen et al. clearly demonstrated that phosphorylation response is tension-dependent [35], this does not preclude spatial or activity regulation of Aurora B as the existing model does not address kinase silencing on correct attachments. Likewise, spatial separation and kinetochore localization need not be mutually exclusive; while spatial separation models were originally based on inner centromere localization of Aurora B, a population located in the inner kinetochore could still be physically separated from outer kinetochore targets under tension. Distance measurements from inner centromere to outer kinetochore suggest these regions may be separated by up to 100 nm [58,126], making the proposed 80 nm dog leash [94] insufficient for Aurora B to reach the outer kinetochore even in tensionless scenarios. If tethered in the inner kinetochore near CENP-C [102] or CENP-A [146], a 80 nm leash would allow Aurora B to reach the outer kinetochore in the absence of tension, but be excluded under tension based on studies that show significant intra-kinetochore stretch: ~20 nm deformation in CENP-C regions [60,75], ~40 nm jack-knifing action in Ndc80, and ~50 nm unraveling of Knl1 [75]. The kinetochore localization model could also be combined with tension-sensitive models; Aurora B activity could be regulated by tension sensors located in the kinetochore. A population of INCENP independent of other CPC members interacts directly with the COMA inner kinetochore sub complex and recruits Aurora B [123,124], and thus could regulate kinase activity at the kinetochore location. In addition to INCENP, Knl1 is a proposed tension-sensitive Aurora B regulator [75]. Phospho-regulation of Aurora B activity in the kinetochore-localized pool has also been demonstrated; only the kinetochore-based pool is phosphorylated by the Chk2 kinase and this phosphorylation confers kinase activity to respond to unattached kinetochores and activate the spindle checkpoint [146,147].

These models provide exciting insights into the translation of mechanical tension force into Aurora B-mediated biochemical signals and offer guidance in future experimentation to distinguish between the different mechanisms. Future experimentation will need to address several outstanding questions, including the activity and localization of Aurora B under tension. One major distinguishing feature between spatial separation-based models and tension-sensitive activation models is the activity status of Aurora B under tension. Previous studies have probed Aurora B activity under tension using phospho-sensors tethered to inner and outer kinetochore proteins and concluded that the kinase is active under tension [44,57]. However, with the discovery of multiple pools of Aurora B [86,101,102,146,147], it is unclear if the kinetochore-based pool responsible for error correction remains active under tension as the other pools’ activity could interfere with these measurements. It will be critical for future experiments to distinguish between these pools, perhaps eliminating the other chromatin-based populations to isolate the error correcting pool for investigation. Likewise, the localization Aurora B has been complicated by these multiple pools as well the limit of imaging resolution in different systems. Determining the precise location of error correcting Aurora B on chromosomes under tension will distinguish between several models, but assessing localization must be measured in live cells with high temporal resolution. The specific tension state of an individual kinetochore can fluctuate with changing attachment type and oscillations on the metaphase spindle [60,152,153].

Future investigations of tension sensing mechanisms will need to address the role of chromosome oscillation in error correction. While not discussed in this review, oscillatory movements of chromosomes on the metaphase spindle are correlated with correction of attachment errors in both mitosis and meiosis (see [154] for a review). Chromosome oscillations are attenuated in cancer cell lines [155] and in cells with non-phosphorylatable Ndc80 [41,151] or deletions of the outer kinetochore protein Dam1 [156]; all of these situations yield errors in attachment correction. Aurora B [41,151] and its family member Aurora A [155], have been shown to phosphorylate sites on Ndc80 that are critical for both chromosome oscillation and error correction. While primarily known for its role in centrosome maturation and cytoskeletal organization (reviewed in [157]), recent studies have revealed a centromeric-based pool of Aurora A [158] and demonstrated its contribution to correcting attachments [155]. Future investigation will need to probe the relationship between the two kinases and determine how Aurora A and chromosome oscillations fit into the models of tension-sensing and error correction.

## Data Availability

Not applicable.
